# The United Nations’ General Assembly High-Level Meeting on antimicrobial resistance: a joint statement from the Heads of Government and Chief Medical Officers of the United Kingdom’s Overseas Territories: Anguilla; Bermuda; British Indian Ocean Territory (BIOT); British Virgin Islands; Cayman Islands; Falkland Islands; Gibraltar; Montserrat; Pitcairn, Henderson, Ducie and Oeno Islands; Saint Helena, Ascension and Tristan da Cunha; South Georgia and the South Sandwich Islands; Turks and Caicos Islands

**DOI:** 10.1093/jac/dkae330

**Published:** 2024-09-19

**Authors:** Matthew Dryden, Michael Corley, Natalie Wright, Aisha Andrewin, Ronald Georges, Shaun Ramroop, Sharra Greenaway, Nicholas Gent, Nadia Astwood, William Hardy, Helen Carter, Peter Moss, Rebecca Edwards, Alex Wonner, Ayo Oyinloye, Kevin Donovan

**Affiliations:** UK Overseas Territories Programme, Global Operations, UK Health Security Agency, London, UK; British Society of Antimicrobial Chemotherapy, 53 Regent Place, Birmingham B1 3NJ, UK; UK Overseas Territories Programme, Global Operations, UK Health Security Agency, London, UK; CMO, Government of Anguilla, Ministry of Health, Anguilla; CMO, Government of BVI, Ministry of Health, British Virgin Islands; CMO, Government of Bermuda, Ministry of Health, Bermuda; CMO, Government of Montserrat, Ministry of Health, Montserrat; CMO Government of Cayman Islands, Ministry of Health, Cayman Islands; CMO, Government of TCI, Ministry of Health, Turks and Caicos Islands; CMO, Government of Ascension Island, Ministry of Health, Ascension Island; CMO Government of Gibraltar, Ministry of Health, Bermuda Gibraltar; CMO Government of St Helena, Ministry of Health, St Helena; CMO, Government of Falkland Islands, Ministry of Health, Falkland Islands; Medical Officer, Camogli Hospital, Tristan da Cunha; CMO, DiegoGarcia, British Indian Ocean Territories; Medical Officer, Pitcairn Islands

## Abstract

The UK Overseas Territories (UKOTs) are small, often remote territories with historical and territorial links to the UK. They range from densely populated areas (Cayman, Bermuda, Gibraltar) to land with no permanent inhabitants (British Antarctic Territory, South Georgia). However, they are linked by ecosystem instability (the permacrisis) including antimicrobial resistance (AMR), climate change and biodiversity disruption. The Chief Medical Officers of the UKOTs met in June 2024 and were unanimous in their concerns about the threat of global AMR. They have issued this statement on their hopes and expectations for the United Nations’ General Assembly High-Level Meeting, in September 2024. These may be summarized by the hope of achieving united and sustained global political will to reduce the threat of AMR by equitable access to treatments, prevention of AMR by sanitation and accurate diagnostics, and education in health care and the public.

## Introduction

The United Nations’ General Assembly High-Level Meeting, in September 2024, offers a rare opportunity for global leaders to agree on a pragmatic approach to containing and controlling the existential threat of antimicrobial resistance (AMR). There is broad agreement that many of the solutions are known—and not beyond what can be afforded if kept simple and done well.

As the representatives of the UK’s 14 Overseas Territories, we call on all Member States of the United Nations to help protect those populations most exposed to the worsening effects of what some experts are calling a ‘permacrisis’, because of AMR’s catastrophic interrelationship with climate change and biodiversity loss.

## The context

1. AMR is one of the world’s biggest killers—with almost 5 million deaths associated with bacterial resistance, including 1.27 million deaths attributable to it. By 2050, at least 10 million people will die from AMR infections every year, costing the global economy $100 trillion. COVID has so far cost the world $17 trillion and is associated with about 3 million deaths. Based on these predictions, the impact of AMR will far exceed that of COVID.
2. AMR hits hardest in low- and middle-income countries (LMICs), where its effects are aggravated by mounting inequity linked to gender, race, age and socioeconomic status. Consequently, AMR poses a significant threat to many of the UN’s 17 Sustainable Development Goals.
3. The threat of AMR is compounded by human behaviour; by our unsustainable treatment of plants (agriculture), animals (livestock—currently, more than 100 000 metric tonnes of antibiotics per year are used in meat production) and the environment, where the spread of AMR is made worse by the impact of climate change, conflict, natural disaster and enforced migration.
4. The United Kingdom Overseas Territories are disproportionally at risk of AMR because of global travel, isolation, geographical position and limited capacities.

## UKOT ambition for the UNGA High-Level Meeting on AMR in 2024

1. The United Nations’ General Assembly commits to creating a political entity, like a Framework Convention, in order to:
  - Secure the interest and support of increasing numbers of UN Member States.
  - Appoint a permanent secretariat to coordinate activity.
  - Enable an independent expert panel to generate and assess scientific evidence and model risks.
  - Agree a monitoring and accountability mechanism to assess progress.
  - Agree, monitor, and refine, targets.
  - Enable catalytic funding—including through the Global Fund. This would see the newly formed secretariat attempt to bring together all available funding and explicitly link its investment to work focused on the targets set by the independent expert panel.
  - Pioneer equitable access to antibiotics and affordable diagnostics.
  - Demonstrate how tackling AMR can also help to mitigate the impact of climate change and biodiversity loss—while considering the corollary of this interrelationship for policy and financing.

## Mid- to long-term aims

1. Build and sustain global political will for action, bolstered by public support, civil society advocacy and private sector partnerships that cross sectors.
2. Ensure all humans, animals and plants, have sustainable and equitable access to appropriate and effective treatments. By doing so, we will:
  - Arrest and reverse mortality and morbidity rates attributable to and associated with AMR, while also containing and controlling rates of resistance.
  - Build health, food and economic security for the world, while ensuring sustainable systems mitigate the impact of AMR on climate change which, in turn, will underpin efforts to achieve the UN’s Sustainable Development Goals.

### Achieving our aims through the three pillars of Access, Prevention, Education

1. ***Access***: Provide access to reliable and cost-effective diagnostics, affordable and appropriate antibiotics, and validated vaccine programs, while securing supply chains, and harvesting emerging innovative technologies. Access must be equitable and sustainable.
2. ***Prevention***: Invest in creditable and sustainable Infection Prevention and Control (IPC) programs in all health care settings. Leverage financial backing for adequate water, sanitation and hygiene (WASH) provision in healthcare facilities. Ensure preventable contamination measures are routinely adopted in farming and aquaculture, and support initiatives to minimize the impact of AMR from human and animal faecal material in communities. Prevention measures should also extend to the implementation of sustainable vaccine programs for both humans and animals
3. ***Education***: Commit to the creation of national and international programs to encourage ‘champions’ in the field of AMR through enhanced medical and veterinary teaching programs, international fellowships and international scholarships. Online learning tools and apps should also play an important role in supporting a more coherent and cohesive approach to education, training and professional development.

This statement was compiled during a conference of the UKOT Chief Medical Officers (the authors) in discussion with the UKOT programme medical team (M.D., N.W.) and officers of the British Society of Antimicrobial Chemotherapy (M.D., M.C.) in June 2024.

## Transparency declarations

None to declare.

